# Entropy Analysis of 3D Non-Newtonian MHD Nanofluid Flow with Nonlinear Thermal Radiation Past over Exponential Stretched Surface

**DOI:** 10.3390/e20120930

**Published:** 2018-12-05

**Authors:** Muhammad Suleman, Muhammad Ramzan, Madiha Zulfiqar, Muhammad Bilal, Ahmad Shafee, Jae Dong Chung, Dianchen Lu, Umer Farooq

**Affiliations:** 1 Department of Mathematics, Faculty of Science, Jiangsu University, Zhenjiang 212013, China; 2 Department of Mathematics, COMSATS University, Islamabad Campus, Islamabad 44000, Pakistan; 3 Department of Computer Science, Bahria University, Islamabad Campus, Islamabad 44000, Pakistan; 4 Department of Mathematics, Allama Iqbal Open University, Islamabad 44000, Pakistan; 5 Department of Mathematics, University of Lahore, Gujrat Campus, Gujranwala 52250, Pakistan; 6 Applied Science Department, College of Technological Studies, Public Authority of Applied Education & Training, Shuwaikh 70030, Kuwait; 7 Department of Mechanical Engineering, Sejong University, Seoul 143-747, Korea

**Keywords:** entropy generation, chemical species, nonlinear thermal radiation, exponential stretched surface

## Abstract

The present study characterizes the flow of three-dimensional viscoelastic magnetohydrodynamic (MHD) nanofluids flow with entropy generation analysis past an exponentially permeable stretched surface with simultaneous impacts of chemical reaction and heat generation/absorption. The analysis was conducted with additional effects nonlinear thermal radiation and convective heat and mass boundary conditions. Apposite transformations were considered to transform the presented mathematical model to a system of differential equations. Analytical solutions of the proposed model were developed via a well-known homotopy analysis scheme. The numerically calculated values of the dimensionless drag coefficient, local Nusselt number, and mass transfer Nusselt number are presented, with physical insights. The graphs depicting the consequences of numerous parameters on involved distributions with requisite deliberations were also a part of this model. It is seen that the Bejan number is an increasing function of the thermal radiation parameter.

## 1. Introduction

Rapid growth in the usage of nanofluids in varied engineering arenas such as cancer therapy, finer coolants in nuclear reactors and computers, numerous electronic devices in military sectors [1], oil and water [2,3], rapid spry cooling, the food industry, vehicles and transformers, polymer extrusion, safe surgeries, quenching in foundries, and glass blowing [4] have encouraged scientists and researchers to scrutinize the numerous aspects of nanofluid flow past different geometries. Nanofluids are an amalgamation of nanoparticles of size < 100 nm (i.e., metals, nitrides, carbides, and nanotubes (single or multiwalled)) and orthodox fluids such as water, toluene, engine, kerosene oil, ethylene and triethylene glycol. Nanofluids are considered to be the best coolants in all engineering applications. It was Choi [5] who experimentally revealed that nanofluids possess enhanced thermal conductivity. Afterward, Buongiorno [6] framed a model pointing out that the heat transfer process is triggered in the case of nanofluids due to thermophoretic diffusion and the Brownian motion of nanoparticles. Later, Khan and Pop [7] discussed nanofluid flow past a stretched surface. Makinde and Aziz supported this model through convective boundary conditions over a stretched surface [8]. The impact of radiative nanoparticle flow with Lorentz force past a spongy semi annulus region was investigated by Sheikholeslami et al. [9]. Li et al. [10] examined the effect of adding nanoparticles in the process of solidification of Nano-enhanced phase change material (NEPCM) in the presence of thermal radiation. The flow of water-based nanofluid containing carbon nanotubes past a permeable medium with the effects of Darcy–Forchheimer was deliberated by Muhammad et al. [11]. Lu et al. [12] pondered on the flow of micropolar nanofluids in the presence of magnetohydrodynamic (MHD), homogeneous-heterogeneous reactions, mixed convection, and thermal radiation past a nonlinear stretched surface, numerically. The combined impacts of Arrhenius activation energy with heat and mass stratification on micropolar nanofluid flow in the presence of binary chemical reactions was studied by Ramzan et al. [13]. Lu et al. [14] did a tremendous study considering the three-layer vertical nanofluid model following a Buongiorno scheme. Li et al. [15], using Finite Element method (FEM), calculated the influence of the transportation of nanoparticles in a permeable enclosure due to electric force. Recently, some studies have discussed the applications of nanofluids in varied fields such as electrokinetic transport [16], heat transfer [17], and mass transfer [18].

The inevitable and dynamic applications of MHD nanofluids (e.g., wound treatment, gastric medications, targeted drug release, sterilized devices, magnetic resonance imagining (MRI), asthma treatment, and removal of tumors with hyperthermia) have attracted the attention of scientists and researchers in studying the related field of nanofluids. Sheikholeslami [19] deliberated the flow of copper oxide-water nanofluids in a spongy channel due to magnetic field effects using the Lattice Boltzmann method (LBM) method. In a recent article, Lu et al. [20] envisaged the model of MHD Carreau nanofluid flow over a radially stretching surface with allied impacts of nonlinear thermal radiation with newly established zero mass flux boundary conditions. They solved the proposed model numerically by engaging the MATLAB bvp4c function. The simultaneous influences of thermal radiation and Cattaneo–Christov heat flux (instead of conventional Fourier’s law of heat conduction) on the MHD nanofluid flow between two parallel plates were examined by Dogonchi and Ganji [21]. They found an analytical solution to the suggested problem by using the Duan–Rach approach (DRA), which facilitates finding undetermined coefficients without the help of numerical techniques. Sheikholeslami [22] deliberated the MHD non-Darcy nanofluid flow in a permeable cubic enclosure using the LBM (lattice Boltzmann method) and highlighted that the Nusselt number is an escalating function of permeability and buoyancy forces. Keeping in mind the applications of nanofluid flow in the attendance of magnetohydrodynamics, some featured recent explorations [23,24,25,26,27] have been added to the literature review.

The study of heat and mass transfer of nanofluid flows with chemical reactions past stretched surfaces has numerous significant applications in core industries such as chemical and metallurgical engineering. Examples include polymer production and food processing. Furthermore, heat and mass transfer models with chemical reaction effects are vital in several processes and have gained attention in recent years. Fascinating applications pertaining to chemical reactions embrace the distribution of temperature and moisture over agricultural fields and groves of fruit trees, the energy transfer of drying and evaporation at the surface of a water body, and the energy transfer of a wet cooling tower.

Entropy is pronounced the nonavailability of the system’s thermal energy for translation into mechanical work. The second law of thermodynamics asserts that throughout the process of conversion of energy into some beneficial work, there is forfeiture of energy that lowers the performance of energy conservation gadgets. Basically, entropy generation is directly proportionate to a loss of energy. Subsequently, entropy generation in a system results in the reduction of the quantity of energy present (exergy). Thus, the efficiency of the thermal system may be enhanced by slashing the entropy generation. In this regard, it is necessary to have the idea of energy generation’s distribution throughout a thermodynamic process with the intention of reduction in entropy generation. The idea of entropy generation was first floated by Bejan [28], who examined the reason for energy generation in a convective heat transfer model. The analysis of entropy generation in viscous fluid flow with an impact of suction and injection past a flat surface was conducted by Reveillere and Baytas [29]. The flow of MHD nanofluid past a spongy narrow vertical channel under the influences of nonlinear thermal radiation, entropy generation, and convective boundary conditions was considered by Lopez et al. [30]. Sheikholeslami [31] deliberated the analysis of exergy and entropy under the influence of Lorentz force past a permeable medium using a new numerical scheme, Control volume finite element method (CVFEM). Some useful recent explorations may be found in References [32,33,34,35,36].

The significance of non-Newtonian fluids cannot be denied due to their involvement in varied engineering and industrial applications such as petroleum production, the thinning of copper wires, and plastic manufacturing [37]. To exhibit such models, Naiver–Stokes equations are not enough since a single constitutive relation does not portray the core requirements of non-Newtonian fluids. That is why several discrete models have been proposed in the literature. Among these, viscoelastic fluid is the differential-type non-Newtonian fluid model that is simplest on its own and exhibits the characteristics of normal stress and shear rate viscosity. Hayat et al. [38] examined the flow of 3D viscoelastic nanofluid over an exponential stretched surface with mixed convection. The 3D viscoelastic nanofluid flow with nonlinear thermal radiation near a stagnation point was discussed by Farooq et al. [39]. Ramzan et al. [40,41,42] deliberated the flow of 3D viscoelastic nanofluid flow with the impacts of Newtonian heating, chemical reaction and MHD, and Soret–Dufour effects. The 3D flow of viscoelastic flow with temperature-dependent thermal conductivity was deliberated by Alsaedi et al. [43]. Some recent attempts considering viscoelastic fluids can be seen in References [44,45].

The literature review above indicates that few studies have been made on three-dimensional viscoelastic nanofluid flows associated with entropy generation and nonlinear thermal radiation past an exponential stretched surface. Therefore, we present our study on such an investigation by solving the proposed model analytically with three dimensionless distributions (Section 2) and examining the effects of different parameters (Section 5).

## 2. Mathematical Modeling

**Figure 1:**
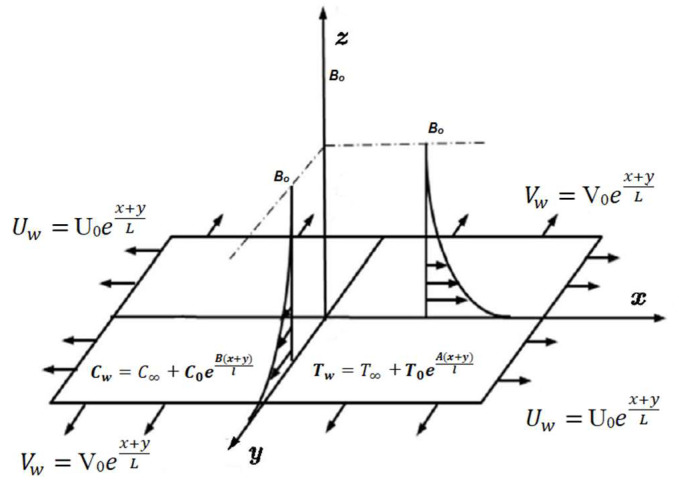
Geometry of the problem.

We considered the flow of MHD viscoelastic nanofluid past a permeable exponentially stretched surface with entropy generation. The analysis was performed in the attendance of nonlinear thermal radiation, chemical reaction, and heat generation and absorption with convective heat and mass boundary conditions. The magnetic field was applied parallel to the *z* axis. The Hall and electric field effects were overlooked. The induced magnetic field was also ignored due to our assumption of a small Reynolds number. The permeable surface was stretched in both the *x* and *y* directions with respective velocities $U_{w}$ and $V_{w}$, as shown in Figure 1.

The mathematical model, under assumptions, is represented by the following equations [46]:

(1) $$\frac{\partial u}{\partial x}+\frac{\partial v}{\partial y}+\frac{\partial w}{\partial z}=\;0,$$

(2) $$\frac{\partial u}{\partial x}+v\frac{\partial u}{\partial y}+w\frac{\partial u}{\partial z}=v(\frac{{\partial^{2}}_{u}}{\partial z^{2}})-k_{0}(u\frac{{\partial^{3}}_{u}}{\partial x\partial z^{2}}+w\frac{{\partial^{3}}_{u}}{\partial z^{3}}-\frac{\partial u}{\partial x}\frac{{\partial^{2}}_{u}}{\partial z^{2}}-\frac{\partial u}{\partial z}\frac{{\partial^{2}}_{w}}{\partial z^{2}}-2\frac{\partial u}{\partial z}\frac{{\partial^{2}}_{u}}{dx\partial z}-2\frac{\partial w}{\partial z}\frac{{\partial^{2}}_{u}}{\partial z^{2}}]-\sigma \frac{\beta 0^{2}}{\rho }u,$$

(3) $$u\frac{\partial v}{\partial x}+v\frac{\partial v}{\partial y}+w\frac{\partial v}{\partial z}=v(\frac{{\partial^{2}}_{v}}{\partial z^{2}})-k_{0}(v\frac{\partial^{3}v}{\partial x\partial z^{2}}+w\frac{{\partial^{3}}_{v}}{\partial z^{3}}-\frac{\partial v}{\partial y}\frac{{\partial^{2}}_{v}}{\partial z^{2}}-\frac{\partial v}{\partial z}\frac{{\partial^{2}}_{w}}{\partial z^{2}}-2\frac{\partial v}{\partial z}\frac{{\partial^{2}}_{v}}{dy\partial z}-2\frac{\partial w}{\partial z}\frac{{\partial^{2}}_{v}}{\partial z^{2}})-\sigma \frac{\beta 0^{2}}{\rho }v,$$

(4) $$u\frac{\partial T}{\partial x}+v\frac{\partial T}{\partial y}+w\frac{\partial T}{\partial z}=\alpha \left(\frac{\partial^{2}T}{\partial z^{2}}\right)+\frac{Q}{\rho c_{f}}\left(T-T_{\infty }\right)+\frac{\left(\rho c_{p}\right)}{\left(\rho c_{f}\right)}\left(D_{B}\left(\frac{\partial T}{\partial z}\frac{\partial C}{\partial z}\right)+\frac{D_{T}}{T_{\infty }}{\left(\frac{\partial T}{\partial z}\right)}^{2}\right)-\frac{1}{\rho c_{p}}\frac{\partial q_{r}}{\partial z},$$

(5) $$u\frac{\partial C}{\partial x}+v\frac{\partial C}{\partial y}+w\frac{\partial C}{\partial z}=\left(D_{B}\left(\frac{\partial^{2}C}{\partial z^{2}}\right)+\frac{D_{T}}{T_{\infty }}\left(\frac{\partial^{2}T}{\partial z^{2}}\right)\right)-K_{c}\left(C-C_{\infty }\right),$$

subjected to the boundary conditions

(6) $$\begin{array}{c} u=U_{w}=U_{0}e^{\frac{x+y}{L}};\;v=V_{w}=V_{0}e^{\frac{x+y}{L}},\;w=0,\\ -k\frac{\partial T}{\partial z}=h_{f}(T_{f}-T),\;-D_{B}\frac{\partial C}{\partial z}=h_{s}(C_{s}-C)\;at\;z=0\\ u\to 0,\;v\to 0,\;T\to T_{\infty },\;C\to C_{\infty }\to \mathrm{as}\to z\to \infty . \end{array}$$

The surface stretching velocities, temperature, and concentration of the wall were given by

(7) $$\begin{array}{c} U_{w}=U_{0}e^{\frac{x+y}{L}};\;V_{w}=V_{0}e^{\frac{x+y}{L}};\;T_{w}=T_{f}=T_{\infty }+T_{0}e^{\frac{A\left(x+y\right)}{L}}\\ C_{w}=\;C_{s}=C_{\infty }+C_{0}e^{\frac{B\left(x+y\right)}{L}}. \end{array}$$

The transformations are presented as given below:

(8) $$\begin{array}{c} u\;=\;U_{0}e^{\frac{x+y}{L}}f^{\prime }\left(\eta \right),\;v=U_{0}e^{\frac{x+y}{L}}g^{\prime }\left(\eta \right),w=-\sqrt{\frac{vU_{0}}{2L}}e^{\frac{x+y}{L}}(f+\eta f^{\prime }+g+\eta g^{\prime }),\\ T\;=T_{\infty }+T_{0}e^{\frac{A\left(x+y\right)}{2L}}\;\theta \left(\eta \right),C\;=C_{\infty }+C_{0}e^{\frac{B\left(x+y\right)}{2L}}\;\phi \left(\eta \right),\eta =\sqrt{\frac{U_{0}}{2vL}}e^{\frac{x+y}{L}}\;z. \end{array}$$

Equation (1) was satisfied automatically, whereas Equations (2)–(5) took the form

(9) $$f^{\prime \prime \prime }+\left(f+g\right)f^{\prime \prime }-2\left(f^{\prime }+g^{\prime }\right)f^{\prime }-K(6f^{\prime \prime \prime }f^{\prime }+\left(3g^{\prime \prime }-3f^{\prime \prime }+\eta g^{\prime \prime \prime }\right)f^{\prime \prime }+\left(4g^{\prime }+2\eta g^{\prime \prime }\right)f^{\prime \prime \prime })-\left(f+g+\eta g^{\prime }\right)f^{⁗})-M^{2}f^{\prime }=0,$$

(10) $$g^{\prime \prime \prime }+\left(f+g\right)g^{\prime \prime }-2\left(f^{\prime }+g^{\prime }\right)g^{\prime }-K(6g^{\prime \prime \prime }g^{\prime }+\left(3f^{\prime \prime }-3g^{\prime \prime }+\eta f^{\prime \prime \prime }\right)g^{\prime \prime }+\left(4f^{\prime }+2\eta f^{\prime \prime }\right)g^{\prime \prime \prime })-\left(f+g+\eta f^{\prime }\right)f^{⁗}-M^{2}g^{\prime }=0,$$

(11) $$\begin{array}{l} \theta^{\prime \prime }+Pr\left((f+g\right)\theta^{\prime }-A\left(f^{\prime }+g^{\prime }\right)\theta +N_{b}\theta^{\prime }\phi^{\prime }+N_{t}{\theta^{\prime }}^{2}+Rd(\frac{4}{3}\theta^{\prime \prime }+4{\left(\theta_{w}-1\right)}^{2}\theta^{2}\theta^{\prime \prime }\\ \;+4\left(\theta_{w}-1\right)\theta \theta^{\prime \prime }+8\left(\theta_{w}-1\right)\theta +8\left(\theta_{w}-1\right)\theta {\theta^{\prime }}^{2}+4{\theta^{\prime }}^{2}+4{\left(\theta_{w}-1\right)}^{2}\theta^{2}{\theta^{\prime }}^{2}\\ \;+\frac{4}{3}{\left(\theta_{w}-1\right)}^{3}\theta^{3}\theta^{\prime \prime }+PrQ\theta =0, \end{array}$$

(12) $$\phi^{\prime \prime }+LePr\left(f+g\right)\phi^{\prime }-BLePr\left(f^{\prime }+g^{\prime }\right)\phi +\frac{N_{t}}{N_{b}}\theta^{\prime \prime }-PrLe\gamma \phi =0,$$

subject to the transformed boundary conditions

(13) $$\begin{array}{c} f=0,\;g=0,\;f^{\prime }=1,g^{\prime }=\beta ,\;\theta^{\prime }=-\;\lambda_{1}\left(1-\theta \left(0\right)\right),\phi^{\prime }=-\;\lambda_{2}\left(1-\phi \left(0\right)\right),at\;\eta =0\\ f^{\prime }\to 0,\;g^{\prime }\to 0,\theta \to 0,\;\phi \to 0\;as\;\eta \to \infty . \end{array}$$

The parameters given in Equations (9)–(12) are defined as follows:

(14) $$\begin{array}{c} K=\frac{k_{0}U_{w}}{2\nu L},\;\beta =\frac{V_{0}}{U_{0}},\;M^{2}=\frac{2\sigma B_{0}{}^{2}L}{\rho U_{w}},\;Pr=\frac{\nu }{\alpha },\;Nb=\frac{{\left(\rho c\right)}_{p}D_{B}(C_{w}-C_{\infty })}{{\left(\rho c\right)}_{f}\nu },\\ N_{t}=\frac{{\left(\rho c\right)}_{p}D_{T}\left(T_{w}-T_{\infty }\right)}{{\left(\rho c\right)}_{f}\nu T_{\infty }},Le=\frac{\alpha }{D_{B}},\;\theta_{w}=\frac{T_{w}}{T_{\infty }},Rd=\frac{4\sigma^{*\;}T_{\infty }{}^{3}}{3k^{*}k_{1}},\;\gamma =\frac{h_{f}}{k}\sqrt{\frac{\nu }{\alpha }}. \end{array}$$

### Skin Friction Coefficient and Local Nusselt and Sherwood Numbers

The equations of the skin friction coefficients along the *x* and *y* directions were given by

(15) $$C_{fx}=\frac{\tau_{wx}}{1/2\rho U_{w}^{2}}=\frac{(µ\frac{\partial u}{\partial z}+\alpha_{1}(u\frac{{\partial^{2}}_{u}}{\partial x\partial z}+v\frac{{\partial^{2}}_{u}}{\partial y\partial z}+w\frac{{\partial^{2}}_{u}}{\partial z^{2}}+\frac{\partial u}{\partial z}\frac{\partial u}{\partial x}+\frac{\partial v}{\partial z}\frac{\partial v}{\partial x}-\frac{\partial w}{\partial z}\frac{\partial u}{\partial z})){|}_{z=0}\;}{1/2\rho U_{w}^{2}},$$

(16) $$C_{fy}=\frac{\tau_{wx}}{1/2\rho U_{w}^{2}}=\frac{(µ\frac{\partial y}{\partial z}+\alpha_{1}(u\frac{{\partial^{2}}_{v}}{\partial x\partial z}+v\frac{{\partial^{2}}_{v}}{\partial y\partial z}+w\frac{{\partial^{2}}_{v}}{\partial z^{2}}+\frac{\partial u}{\partial z}\frac{\partial u}{\partial y}+\frac{\partial v}{\partial z}\frac{\partial v}{\partial y}-\frac{\partial w}{\partial z}\frac{\partial v}{\partial z})){|}_{z=0}\;}{1/2\rho U_{w}^{2}}.$$

The equations of the skin friction coefficients in dimensionless form were given by

(17) $$C_{fx}={\left(\frac{Re}{2}\right)}^{-\frac{1}{2}}(f^{\prime \prime }+K\left(-\left(f+g\right)f^{\prime \prime \prime }+5\left(f^{\prime }+g^{\prime }\right)f^{\prime \prime }+2f^{\prime }f^{\prime \prime }+2g^{\prime }g^{\prime \prime }\right)){|}_{\eta =0},$$

(18) $$C_{fy}={\left(\frac{Re}{2}\right)}^{-\frac{1}{2}}{(}^{\prime \prime }+K\left(-\left(f+g\right)f^{\prime \prime \prime }+5\left(f^{\prime }+g^{\prime }\right)g^{\prime \prime }+2f^{\prime }f^{\prime \prime }+2g^{\prime }g^{\prime \prime }\right)){|}_{\eta =0},$$

and the rates of heat and mass transfers in dimensionless forms were appended

(19) $$Nu_{x}=-\frac{x}{\left(T_{w}-T_{\infty }\right)}\left(\frac{\partial T}{\partial z}\right){|}_{z=0}=-\frac{x}{L}{\left(\frac{Re}{2}\right)}^{\frac{1}{2}}\theta^{\prime }\left(0\right),$$

(20) $$Sh_{x}=-\frac{x}{\left(C_{w}-C_{\infty }\right)}\left(\frac{\partial C}{\partial z}\right){|}_{z=0}=-\frac{x}{L}{\left(\frac{Re}{2}\right)}^{\frac{1}{2}}\phi^{\prime }\left(0\right).$$

## 3. Convergence Analysis

**Figure 2:**
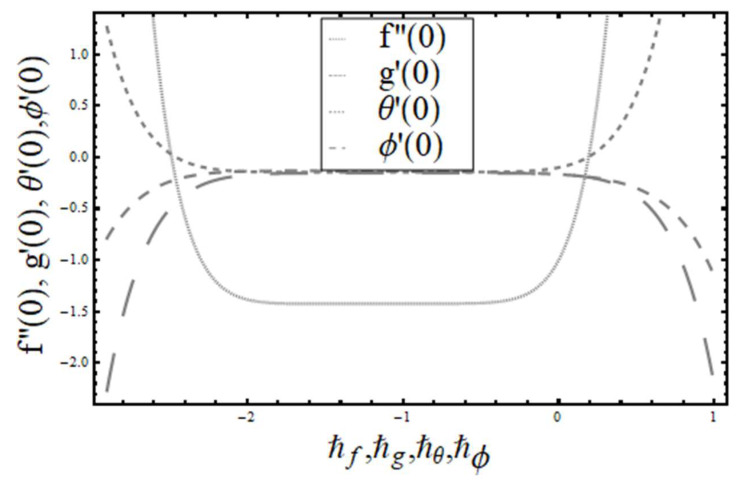
The *ħ* curves for f,g,θ,ϕ.

The convergence of the series solutions was adjusted and regulated with the aid of auxiliary parameters $\hbar_{f},\hbar_{g},\hbar_{\theta }$, and $\hbar_{\emptyset }$. For tolerable ranges, ℏ-curves were drawn and are given in Figure 2. These admissible ranges were $\hbar_{f},\hbar_{g},\hbar_{\theta }$, and $\hbar_{\phi }$, which were $-1.85\ll \hbar_{f}\ll -0.70,$ $-1.85\ll \hbar_{f}\ll -0.70,$ $-1.80\ll \hbar_{g}\ll -0.90,$ $-1.80\ll \hbar_{\theta }\ll -0.70,$ and $-1.80\ll \hbar_{\phi }\ll -0.70.$ These values were corroborated by the numerical values given in Table 1.

### 3.1. Homotopic Solutions

The homotopy analysis method necessitated the initial guesstimates ($f_{0}$, $g_{0}$, $\theta_{0},\phi_{0}$) with auxiliary linear operators ($L_{f}$, $L_{g},L_{\theta },L_{\phi }$) in the form [43]

(21) $$f_{0}\left(\eta \right)\;=1-e^{-\eta },g_{0}(\eta )=\lambda (1-e^{-\eta }),\theta_{0}\left(\eta \right)=\frac{\gamma }{1+\gamma }e^{-\eta },\;\phi_{0}\left(\eta \right)=\frac{\gamma }{1+\gamma }e^{-\eta },$$

(22) $$L_{f}=f^{\prime \prime \prime }-f^{\prime },L_{g}=g^{\prime \prime \prime }-g^{\prime },L_{\theta }=\theta^{\prime \prime }-\theta ,L_{\phi }=\phi^{\prime \prime }-\phi$$

These auxiliary linear operators possessed the ensuing features

(23) $$\begin{array}{c} L_{f}\left(B_{1}+B_{2}e^{\eta }+B_{3}e^{-\eta }\right)=0,L_{g}\left(B_{4}+B_{5}e^{\eta }+B_{6}e^{-\eta }\right)=0,\\ L_{\theta }\left(B_{7}e^{\eta }+B_{8}e^{-\eta }\right)=0,L_{\phi }\left(B_{9}e^{\eta }+B_{10}e^{-\eta }\right)=0, \end{array}$$

where $B_{i}$, i=1−10 are arbitrary constants.

### 3.2. Deformation Problems at Zeroth Order

Here,

(24) $$\left(1-p\right)L_{f}\left(\hat{f}\left(\eta ;p\right)-f_{0}\left(\eta \right)\right)=p\hbar_{f}N_{f}\left(\hat{f}\left(\eta ;p\right),\hat{g}\left(\eta ;p\right)\right),$$

(25) $$\left(1-p\right)L_{g}\left(\hat{g}\left(\eta ;p\right)-g_{0}\left(\eta \right)\right)=p\hbar_{g}N_{g}\left(\hat{f}\left(\eta ;p\right),\hat{g}\left(\eta ;p\right)\right),$$

(26) $$\left(1-p\right)L_{\theta }\left(\hat{\theta }\left(\eta ;p\right)-\theta_{0}\left(\eta \right)\right)=p\hbar_{\theta }N_{\theta }\left(\hat{f}\left(\eta ;p\right),\hat{g}\left(\eta ;p\right),\hat{\theta }\left(\eta ;p\right),\hat{\phi }\left(\eta ;p\right)\right),$$

(27) $$\left(1-p\right)L_{\phi }\left(\hat{\phi }\left(\eta ;p\right)-\phi_{0}\left(\eta \right)\right)=p\hbar_{\phi }N_{\phi }\left(\hat{f}\left(\eta ;p\right),\hat{g}\left(\eta ;p\right),\hat{\theta }\left(\eta ;p\right),\hat{\phi }\left(\eta ;p\right)\right),$$

(28) $$\hat{g}\left(0;p\right)=0,{\hat{f}}^{\prime }\left(\infty ;p\right)=0,{\hat{g}}^{\prime }\left(\infty ;p\right)=0,$$

(29) $$\hat{g}\left(0;p\right)=0,{\hat{g}}^{\prime }\left(0;p\right)=\beta ,{\hat{g}}^{\prime }\left(\infty ;p\right)=0,$$

(30) $$\hat{\theta }\left(0;p\right)=-\gamma \left(1-\hat{\theta }\left(0;p\right)\right),\hat{\theta }\left(\infty ;p\right)=0,\phi \left(0;p\right)=Nb\hat{\phi }\left(0;p\right)+Nt\hat{\theta }\left(0;p\right),\phi \left(\infty ;p\right)=0,$$

where $\hbar_{f},\hbar_{g},\hbar_{\theta }$, and $\hbar_{\phi }$ characterize the nonzero convergence control parameters and p∈[0,1] specifies the embedding parameter, whereas the nonlinear operators $N_{f},N_{g},N_{\theta }$, and $N_{\phi }$ are specified by

(31) $$N_{f}\left(\hat{f}\left(\eta ;p\right),\hat{g}\left(\eta ;p\right)\right)=\frac{\partial^{3}\hat{f}\left(\eta ;p\right)}{\partial \eta^{3}}-M^{2}\frac{\partial \hat{f}\left(\eta ;p\right)}{\partial \eta }+\left(\hat{f}\left(\eta ,p\right)+\hat{g}\left(\eta ,p\right)\right)\frac{\partial^{2}\hat{f}\left(\eta ;p\right)}{\partial \eta^{2}}-\;2\left(\frac{\partial \hat{f}\left(\eta ;p\right)}{\partial \eta }+\frac{\partial \hat{g}\left(\eta ,p\right)}{\partial \eta }\right)\frac{\partial \hat{f}\left(\eta ,p\right)}{\partial \eta }-\;K(6\frac{\partial \hat{f}\left(\eta ,p\right)}{\partial \eta }\frac{\partial^{3}f\left(\eta ,p\right)}{\partial \eta^{3}}+\left\{3\frac{\partial^{2}\hat{g}\left(\eta ,p\right)}{\partial \eta^{2}}-3\frac{\partial^{2}\hat{f}\left(\eta ,p\right)}{\partial \eta^{2}}-3\frac{\partial^{2}\hat{f}\left(\eta ,p\right)}{\partial \eta^{2}}+\eta \frac{\partial^{3}\hat{g}\left(\eta ,p\right)}{\partial \eta^{3}}\right\}\frac{\partial^{2}\hat{f}\left(\eta ;p\right)}{\partial \eta^{2}}+\;\left\{4\frac{\partial \hat{g}\left(\eta ,p\right)}{\partial \eta }+2\eta \frac{\partial^{2}\hat{g}\left(\eta ,p\right)}{\partial \eta^{2}}\right\}\frac{\partial^{3}\hat{f}\left(\eta ;p\right)}{\partial \eta^{3}})-\left(\hat{f}\left(\eta ,p\right)+\hat{g}\left(\eta ,p\right)+\eta \frac{\partial \hat{g}\left(\eta ,p\right)}{\partial \eta }\right)\frac{\partial^{4}\hat{f}\left(\eta ;p\right)}{\partial \eta^{4}},$$

(32) $$N_{g}\left(\hat{g}\left(\eta ;p\right),\hat{g}\left(\eta ;p\right)\right)=\frac{\partial^{3}\hat{g}\left(\eta ;p\right)}{\partial \eta^{3}}-M^{2}\frac{\partial \hat{g}\left(\eta ;p\right)}{\partial \eta }+\left(\hat{f}\left(\eta ,p\right)+\hat{g}\left(\eta ,p\right)\right)\frac{\partial^{2}\hat{g}\left(\eta ;p\right)}{\partial \eta^{2}}-\;2\left(\frac{\partial \hat{f}\left(\eta ;p\right)}{\partial \eta }+\frac{\partial \hat{g}\left(\eta ,p\right)}{\partial \eta }\right)\frac{\partial \hat{g}\left(\eta ,p\right)}{\partial \eta }-K(6\frac{\partial \hat{g}\left(\eta ,p\right)}{\partial \eta }\frac{\partial^{3}\hat{g}\left(\eta ,p\right)}{\partial \eta^{3}}+\;\left\{3\frac{\partial^{2}\hat{f}\left(\eta ,p\right)}{\partial \eta^{2}}-3\frac{\partial^{2}\hat{g}\left(\eta ,p\right)}{\partial \eta^{2}}+\eta \frac{\partial^{3}\hat{f}\left(\eta ,p\right)}{\partial \eta^{3}}\right\}\frac{\partial^{2}\hat{g}\left(\eta ;p\right)}{\partial \eta^{2}}+4\left\{\frac{\partial \hat{f}\left(\eta ,p\right)}{\partial \eta }+2\eta \frac{\partial^{2}\hat{f}\left(\eta ,p\right)}{\partial \eta^{2}}\right\}\frac{\partial^{3}\hat{g}\left(\eta ;p\right)}{\partial \eta^{3}})-\;\left(\hat{f}\left(\eta ,p\right)+\hat{g}\left(\eta ,p\right)+\eta \frac{\partial \hat{f}\left(\eta ,p\right)}{\partial \eta }\right)\frac{\partial^{4}\hat{g}\left(\eta ;p\right)}{\partial \eta^{4}},$$

(33) $$N_{\theta }\left(\hat{f}\left(\eta ;p\right),\hat{g}\left(\eta ;p\right),\hat{\theta }\left(\eta ,p\right),\hat{\phi }\left(\eta ,p\right)\right)=\frac{\partial^{2}\hat{\theta }\left(\eta ,p\right)}{\partial \eta^{2}}+Pr\left(\hat{f}\left(\eta ,p\right)+\hat{g}\left(\eta ,p\right)\right)\frac{\partial \hat{\theta }\left(\eta ,p\right)}{\partial \eta }\;-APr\left(\frac{\partial \hat{f}\left(\eta ;p\right)}{\partial \eta }+\frac{\partial \hat{g}\left(\eta ,p\right)}{\partial \eta }\right)\hat{\theta }\left(\eta ,p\right)+NbPr\left(\frac{\partial \hat{\theta }\left(\eta ,p\right)}{\partial \eta }\frac{\partial \hat{\phi }\left(\eta ,p\right)}{\partial \eta }\right)+NtPr{\left(\frac{\partial \hat{\theta }\left(\eta ,p\right)}{\partial \eta }\right)}^{2}\;+\frac{4}{3}Rd{\left(\theta_{w}-1\right)}^{3}{\hat{\theta }}^{3}\left(\eta ,p\right)\frac{\partial^{2}\hat{\theta }\left(\eta ,p\right)}{\partial \eta^{2}}+\frac{4}{3}Rd\frac{\partial^{2}\hat{\theta }\left(\eta ,p\right)}{\partial \eta^{2}}+4Rd\left(\theta_{w}-1\right){\hat{\theta }}^{2}\left(\eta ,p\right)\frac{\partial^{2}\hat{\theta }\left(\eta ,p\right)}{\partial \eta^{2}}\;+4Rd\left(\theta_{w}-1\right)\hat{\theta }\left(\eta ,p\right)\frac{\partial^{2}\hat{\theta }\left(\eta ,p\right)}{\partial \eta^{2}}+8Rd\left(\theta_{w}-1\right)\hat{\theta }\left(\eta ,p\right){\left(\frac{\partial \hat{\theta }\left(\eta ,p\right)}{\partial \eta }\right)}^{2}+\;+4Rd{\left(\frac{\partial \hat{\theta }\left(\eta ,p\right)}{\partial \eta }\right)}^{2}+4Rd{\left(\theta_{w}-1\right)}^{2}{\hat{\theta }}^{2}\left(\eta ,p\right){\left(\frac{\partial \hat{\theta }\left(\eta ,p\right)}{\partial \eta }\right)}^{2}+PrQ\hat{\theta }\left(\eta ,p\right),$$

(34) $$N_{\phi }\left(\hat{f}\left(\eta ;p\right),\hat{g}\left(\eta ;p\right),\hat{\theta }\left(\eta ,p\right),\hat{\phi }\left(\eta ,p\right)\right)=\frac{\partial^{2}\hat{\phi }\left(\eta ,p\right)}{\partial \eta^{2}}+LePr\left(\hat{f}\left(\eta ,p\right)+\hat{g}\left(\eta ,p\right)\right)\;\frac{\partial \hat{\phi }\left(\eta ,p\right)}{\partial \eta }-BLePr\left(\frac{\partial \hat{f}\left(\eta ,p\right)}{\partial \eta }+\frac{\partial \hat{g}\left(\eta ,p\right)}{\partial \eta }\right)\hat{\phi }\left(\eta ,p\right)+\frac{Nt}{Nb}\frac{\partial^{2}\hat{\theta }\left(\eta ,p\right)}{\partial \eta^{2}}\;+\frac{Nt}{Nb}\frac{\partial^{2}\hat{\theta }\left(\eta ,p\right)}{\partial \eta^{2}}-PrLe\hat{\phi }\left(\eta ,p\right).$$

For p=0 and p=1, we get

(35) $$\begin{array}{l} \hat{f}\left(\eta ;0\right)=f_{0}\left(\eta \right),\hat{g}\left(\eta ;0\right)=g_{0}\left(\eta \right),\hat{\theta }\left(\eta ;0\right)=\theta_{0}\left(\eta \right),\hat{\phi }\left(\eta ;0\right)=\phi_{0}\left(\eta \right)-\gamma \left(1-\hat{\theta }\left(\eta ;0\right)\right),\\ \hat{\phi }\left(\eta ;0\right)=Nb\hat{\phi }\left(\eta ;0\right)+Nt\hat{\theta }\left(\eta ;0\right),\hat{f}\left(\eta ;1\right)=f\left(\eta \right),\hat{g}\left(\eta ;1\right)=g\left(\eta \right),\hat{\theta }\left(\eta ;1\right)=\theta \left(\eta \right),\hat{\phi }\left(\eta ;1\right)=\phi \left(\eta \right) \end{array}$$

The subsequent expressions were attained via Taylor’s series expansion:

(36) $$\hat{f}\left(\eta ;p\right)=f_{0}\left(\eta \right)+\sum_{m=1}^{\infty }f_{m}p^{m},f_{m}\left(\eta \right)={\frac{1}{m!}\frac{\partial^{m}\hat{f}\left(\eta ;p\right)}{\partial p^{m}}|}_{p=0},$$

(37) $$\hat{g}\left(\eta ;p\right)=g_{0}\left(\eta \right)+\sum_{m=1}^{\infty }g_{m}p^{m},g_{m}\left(\eta \right)={\frac{1}{m!}\frac{\partial^{m}\hat{g}\left(\eta ;p\right)}{\partial p^{m}}|}_{p=0},$$

(38) $$\hat{\theta }\left(\eta ;p\right)=\theta_{0}\left(\eta \right)+\sum_{m=1}^{\infty }\theta_{m}\left(\eta \right)p^{m},\theta_{m}\left(\eta \right)={\frac{1}{m!}\frac{\partial^{m}\hat{\theta }\left(\eta ;p\right)}{\partial p^{m}}|}_{p=0},$$

(39) $$\hat{\phi }\left(\eta ;p\right)=\phi_{0}\left(\eta \right)+\sum_{m=1}^{\infty }\phi_{m}\left(\eta \right)p^{m},\phi_{m}\left(\eta \right)={\frac{1}{m!}\frac{\partial^{m}\phi \left(\eta ;p\right)}{\partial p^{m}}|}_{p=0}.$$

The convergence of control parameters $\hbar_{f},\hbar_{g},\;\hbar_{\theta }$, and $\hbar_{\phi }$ were selected in such a way that the series (36)–(39) converged at p=1. Then

(40) $$\hat{f}(\eta ,p)=f_{0}(\eta )+\sum_{m=1}^{\infty }f_{m}(\eta ),$$

(41) $$\hat{g}\left(\eta ;p\right)=g_{0}\left(\eta \right)+\sum_{m=1}^{\infty }g_{m}\left(\eta \right),$$

(42) $$\hat{\theta }\left(\eta ;p\right)=\theta_{0}\left(\eta \right)+\sum_{m=1}^{\infty }\theta_{m}\left(\eta \right),$$

(43) $$\hat{\phi }\left(\eta ;p\right)=\phi_{0}\left(\eta \right)+\sum_{m=1}^{\infty }\phi_{m}\left(\eta \right).$$

### 3.3. The m-th Order Problem

The equations of the *m*-th order are

(44) $$L_{f}\left(f_{m}\left(\eta \right)-X_{m}f_{m-1}\left(\eta \right)\right)=\hbar_{f}R_{f}^{m}\left(\eta \right),$$

(45) $$L_{g}\left(g_{m}\left(\eta \right)-X_{m}g_{m-1}\left(\eta \right)\right)=\hbar_{g}R_{g}^{m}\left(\eta \right),$$

(46) $$L_{\theta }\left(\theta_{m}\left(\eta \right)-X_{m}\theta_{m-1}\left(\eta \right)\right)=\hbar_{\theta }R_{\theta }^{m}\left(\eta \right),$$

(47) $$L_{\phi }\left(\phi_{m}\left(\eta \right)-X_{m}\phi_{m-1}\left(\eta \right)\right)=\hbar_{\phi }R_{\phi }^{m}\left(\eta \right),$$

(48) $$R_{f}^{m}\left(\eta \right)=f_{m-1}^{\prime \prime \prime }\left(\eta \right)-M^{2}f_{m-1}^{\prime }\left(\eta \right)+\sum_{k=0}^{m-1}\left(f_{m-1-k}f_{k}^{\prime \prime }+g_{m-1-k}^{\prime }f_{k}^{\prime \prime }\right)-2\sum_{k=0}^{m-1}f_{m-1-k}^{\prime }f_{k}^{\prime }\;-2\sum_{k=0}^{m-1}g_{m-1-k}^{\prime }f_{k}^{\prime }-k(6\sum_{k=0}^{m-1}f_{m-1-k}^{\prime }f_{k}^{\prime \prime \prime }+3\sum_{k=0}^{m-1}g_{m-1-k}^{\prime \prime }f_{k}^{\prime \prime }-3\sum_{k=0}^{m-1}f_{m-1-k}^{\prime \prime }f_{k}^{\prime \prime }+\sum_{k=0}^{m-1}\eta g_{m-1-k}^{\prime \prime \prime }f_{k}^{\prime \prime }\;+4\sum_{k=0}^{m-1}g_{m-1-k}^{\prime }f_{k}^{\prime \prime \prime }+2\sum_{k=0}^{m-1}\eta g_{m-1-k}^{\prime \prime }f_{k}^{\prime \prime \prime }-\sum_{k=0}^{m-1}f_{m-1-k}f_{k}^{⁗}-\sum_{k=0}^{m-1}\eta g_{m-1-k}^{\prime }f^{⁗}),$$

(49) $$R_{g}^{m}\left(\eta \right)=g_{m-1}^{\prime \prime \prime }\left(\eta \right)-M^{2}g_{m-1}^{\prime }\left(\eta \right)+\sum_{k=0}^{m-1}\left(f_{m-1-k}g_{k}^{\prime \prime }+g_{m-1-k}^{\prime }g_{k}^{\prime \prime }\right)-2\sum_{k=0}^{m-1}g_{m-1-k}^{\prime }g_{k}^{\prime }\;-2\sum_{k=0}^{m-1}g_{m-1-k}^{\prime }f_{k}^{\prime }-k(6\sum_{k=0}^{m-1}g_{m-1-k}^{\prime }f_{k}^{\prime \prime \prime }+3\sum_{k=0}^{m-1}f_{m-1-k}^{\prime \prime }g_{k}^{\prime \prime }-3\sum_{k=0}^{m-1}g_{m-1-k}^{\prime \prime }g_{k}^{\prime \prime }+\sum_{k=0}^{m-1}\eta f_{m-1-k}^{\prime \prime \prime }g_{k}^{\prime \prime }\;+4\sum_{k=0}^{m-1}f_{m-1-k}^{\prime }g_{k}^{\prime \prime \prime }+2\sum_{k=0}^{m-1}\eta f_{m-1-k}^{\prime \prime }g_{k}^{\prime \prime \prime }-\sum_{k=0}^{m-1}g_{m-1-k}g_{k}^{⁗}-\sum_{k=0}^{m-1}\eta f_{m-1-k}^{\prime }g^{⁗}),$$

(50) $$R_{\theta }^{m}\left(\eta \right)=\theta_{m-1}^{\prime \prime }\left(\eta \right)+Pr\sum_{k=0}^{m-1}\left(f_{m-1-k}\theta_{k}^{\prime }+g_{m-1-k}\theta_{k}^{\prime }\right)-APr\sum_{k=0}^{m-1}\left(f_{m-1-k}^{\prime }\theta_{k}+g_{m-1-k}^{\prime }\theta_{k}\right)\;+NbPr\sum_{k=0}^{m-1}\theta_{m-1-k}^{\prime }\phi_{k}^{\prime }+NtPr\sum_{k=0}^{m-1}\theta_{m-1-k}^{\prime }\theta_{k}^{\prime }+Rd(\frac{4}{3}{\left(\theta_{w}-1\right)}^{3}\sum_{k=0}^{m-1}\theta_{m-1-k}^{3}\theta_{k}^{\prime \prime }+\;\frac{4}{3}\theta_{m-1}^{\prime \prime }\left(\eta \right)+4{\left(\theta_{w}-1\right)}^{2}\sum_{k=0}^{m-1}\theta_{m-1-k}^{2}\theta_{k}^{\prime \prime }+4\left(\theta_{w}-1\right)\sum_{k=0}^{m-1}\theta_{m-1-k}\theta_{k}^{\prime \prime }+\;8\left(\theta_{w}-1\right)\sum_{k=0}^{m-1}\theta_{m-1-k}\theta_{k}^{\prime \prime }+4\theta_{m-1}^{\prime }\left(\eta \right)+4{\left(\theta_{w}-1\right)}^{2}\sum_{k=0}^{m-1}\theta_{m-1-k}^{2}\theta_{k}^{\prime^{2}})+PrQ\theta_{m-1}\left(\eta \right),$$

(51) $$R_{\phi }^{m}\left(\eta \right)=\phi_{m-1}^{\prime \prime }\left(\eta \right)+LePr\sum_{k=0}^{m-1}\left(f_{m-1-k}\phi_{k}^{\prime }-g_{m-1-k}\phi_{k}^{\prime }\right)-BLePr,$$

(52) $$X_{m}=\left\{ \begin{array}{l} 0m\leq 1\\ 1m>1 \end{array}\right..$$

The general solutions $\left(f_{m},g_{m},\theta_{m},\phi_{m}\right)$ for Equations (48)–(51) in terms of special solutions $\left({f^{*}}_{m},{g^{*}}_{m},{\theta^{*}}_{m},{\phi^{*}}_{m}\right)$ are given by

(53) $$f_{m}\left(\eta \right)=f_{m}^{*}\left(\eta \right)+C_{1}+C_{2}e^{\eta }+C_{3}e^{-\eta },$$

(54) $$g_{m}\left(\eta \right)=g_{m}^{*}\left(\eta \right)+C_{4}+C_{5}e^{\eta }+C_{6}e^{-\eta },$$

(55) $$\theta_{m}\left(\eta \right)=\theta_{m}^{*}\left(\eta \right)+C_{7}e^{\eta }+C_{8}e^{-\eta },$$

(56) $$\phi_{m}\left(\eta \right)=\phi_{m}^{*}\left(\eta \right)+C_{9}e^{\eta }+C_{10}e^{-\eta }.$$

## 4. Entropy Analysis

The equation of entropy generation [47,48,49] was given by

(57) $$\begin{array}{ll} S_{G} & =\underset{a}{\underbrace{\frac{K}{{T_{\infty }}^{2}}\left(1+\frac{16\sigma^{*}T^{3}}{3KK^{*}}\right){\left(\frac{\partial T}{\partial z}\right)}^{2}}}+\underset{b}{\underbrace{\frac{RD_{B}}{C_{\infty }}{\left(\frac{\partial C}{\partial z}\right)}^{2}+\frac{RD_{B}}{T_{\infty }}\left(\frac{\partial C}{\partial z}\frac{\partial T}{\partial z}\right)}}\\ & +\underset{c}{\underbrace{\frac{\rho C_{p}}{T_{\infty }}D}}+\underset{d}{\underbrace{\frac{\sigma {B_{0}}^{2}}{T_{\infty }}\left(u^{2}+v^{2}\right)}}, \end{array}$$

where *a*, *b*, *c* and *d* represent the thermal irreversibility or entropy generation due to heat transfer, the concentration irreversibility or entropy generation due to mass transfer, the entropy generation due to viscous effects in the fluid, and the entropy generation due to magnetic effects of the fluid, respectively. Then

(58) $$D={\left(\frac{\partial C}{\partial z}\right)}^{2}\left\{\begin{array}{l} \left(\frac{\partial u}{\partial x}\right)\left(\frac{2\nu }{C_{p}\left(1+\lambda_{1}\right)}\left(\frac{\partial u}{\partial x}\right)+\frac{2\nu \lambda_{2}}{C_{p}\left(1+\lambda_{1}\right)}\left(u\frac{\partial^{2}u}{\partial x^{2}}+v\frac{\partial^{2}u}{\partial x\partial y}+w\frac{\partial^{2}u}{\partial x\partial z}\right)\right)+\\ \left(\frac{\partial v}{\partial y}\right)\left(\frac{2\nu }{C_{p}\left(1+\lambda_{1}\right)}\left(\frac{\partial v}{\partial x}\right)+\frac{2\nu \lambda_{2}}{C_{p}\left(1+\lambda_{1}\right)}\left(u\frac{\partial^{2}v}{\partial x\partial y}+v\frac{\partial^{2}v}{\partial^{2}y}+w\frac{\partial^{2}v}{\partial y\partial z}\right)\right) \end{array}\right\},$$

and

(59) $${\left(S_{gen}\right)}_{0}=\frac{K}{T_{\infty }{}^{2}}{\left(\frac{∆T}{L^{2}}\right)}^{2}$$

is the characteristic entropy generation rate. Using the transformations in Equation (6), Equation (21) takes the form

(60) $$\begin{array}{ll} Ns= & \frac{S_{gen}}{{\left(S_{gen}\right)}_{0}}=\frac{Re}{2}e^{\chi }\left(\left(1+\frac{4Rd}{3}{\left(\theta \left(\theta_{w}-1\right)+1\right)}^{3}\right){\theta^{\prime }}^{2}+\frac{\sum^{2}\varepsilon }{\Omega^{2}}{\phi^{\prime }}^{2}+\frac{\sum \varepsilon }{\Omega }\theta^{\prime }\phi^{\prime }\right)\\ & +\frac{Bre^{\chi }}{\Omega \left(1+\lambda_{1}\right)}d+\frac{MBr}{\Omega }{f^{\prime }}^{2} \end{array}$$

where

(61) $$\begin{array}{l} d=\left(2f^{\prime }+\eta f^{\prime \prime }\right)\left(\left(2f^{\prime }+\eta f^{\prime \prime }\right)+\frac{\beta S}{2}\left(\left(4f^{\prime }+2\eta f^{\prime \prime }\right)\left(f^{\prime }+g^{\prime }\right)-\left(3f^{\prime \prime }+\eta f^{\prime \prime \prime }\right)\left(f+g\right)\right)\right)+\\ \left(2g^{\prime }+\eta g^{\prime \prime }\right)\left(\left(2g^{\prime }+\eta g^{\prime \prime }\right)+\frac{\beta S}{2}\left(\left(4g^{\prime }+2\eta g^{\prime \prime }\right)\left(f^{\prime }+g^{\prime }\right)-\left(3g^{\prime \prime }+\eta g^{\prime \prime \prime }\right)\left(f+g\right)\right)\right) \end{array}$$

An important Bejan number defined by the ratio of entropy generation due to thermal effect and total entropy generated by thermal, concentration, and fluid frictions forces was as follows [50]:

(62) $$Be=\frac{\mathrm{The}\;\mathrm{entropy}\;\mathrm{generation}\;\mathrm{due}\;\mathrm{to}\;\mathrm{thermal}\;\mathrm{irreversibility}}{\mathrm{The}\;\mathrm{total}\;\mathrm{entropy}\;\mathrm{generation}}.$$

In dimensionless form, it became

(63) $$Be=\frac{\frac{Re}{2}e^{\chi }\left(1+\frac{4Rd}{3}{\left(\theta \left(\theta_{w}-1\right)+1\right)}^{3}\right){\theta^{\prime }}^{2}}{\frac{Re}{2}e^{\chi }\left(\left(1+\frac{4Rd}{3}{\left(\theta \left(\theta_{w}-1\right)+1\right)}^{3}\right){\theta^{\prime }}^{2}+\frac{\sum^{2}\varepsilon }{\Omega^{2}}{\phi^{\prime }}^{2}+\frac{\sum \varepsilon }{\Omega }\theta^{\prime }\phi^{\prime }\right)+\frac{Bre^{\chi }}{\Omega \left(1+\lambda_{1}\right)}d+\frac{MBr}{\Omega }{f^{\prime }}^{2}}.$$

The value of the irreversibility parameter must lie between 0<Be<1. if Be=0, then there is no entropy generated due to heat transfer. When Be<0.5, then the entropy generation due to fluid friction is dominant over the entropy generation due to heat transfer. On the other hand, if Be>0, then reverse relation is obvious for the entropy generation due to heat and total entropy generation.

The averaged entropy generation number could be evaluated using the following integral formula:

(64) $${\left[Ns\right]}_{avg}=\frac{1}{\forall }\int_{\forall }Nsd\forall ,$$

where ∀ is the length of the boundary layer region.

## 5. Results and Discussion

**Figure 3:**
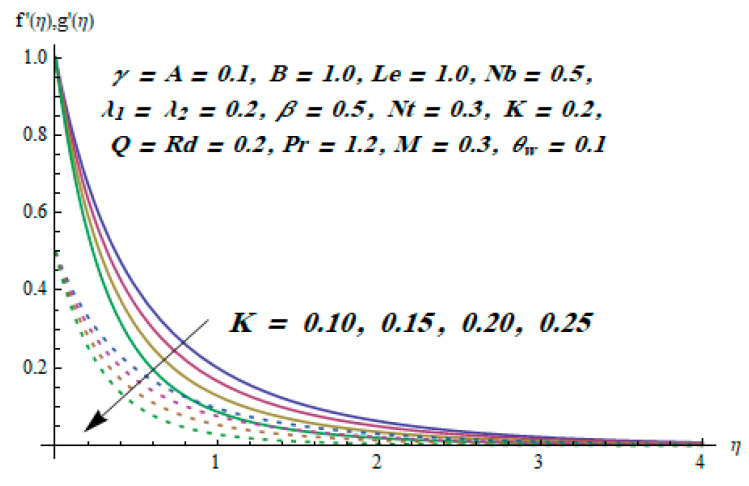
Effect of *K* on $f^{\prime }\;\mathrm{and}\;g^{\prime }$.

**Figure 4:**
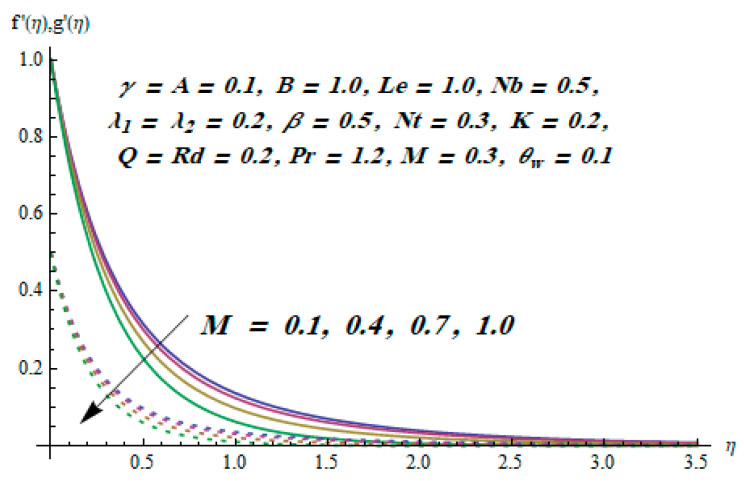
Effect of *M* on $f^{\prime }$ and $g^{\prime }$.

**Figure 5:**
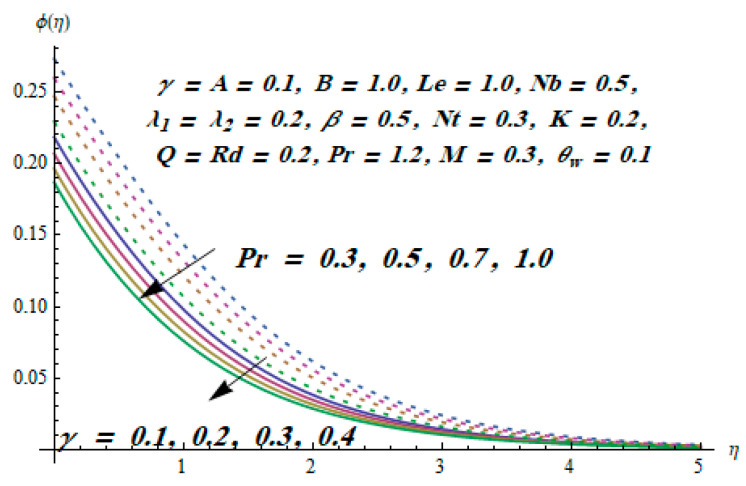
Effect of *Pr* and *γ* on ϕ.

**Figure 6:**
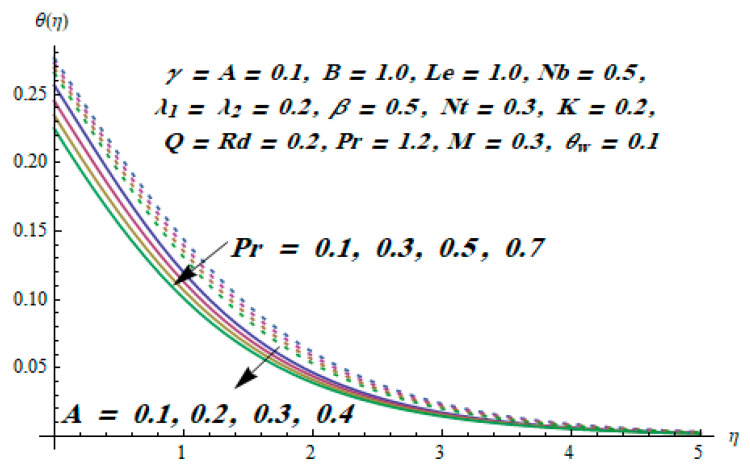
Effect of *Pr* and *A* on θ.

**Figure 7:**
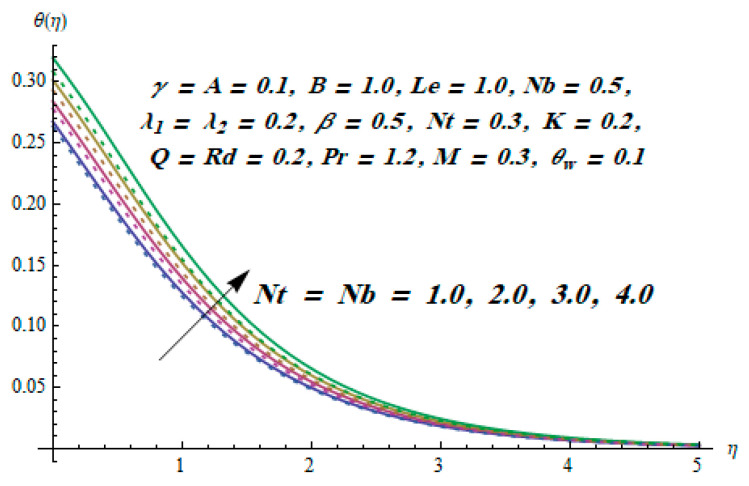
Effect of Nt and Nb on  θ.

**Figure 8:**
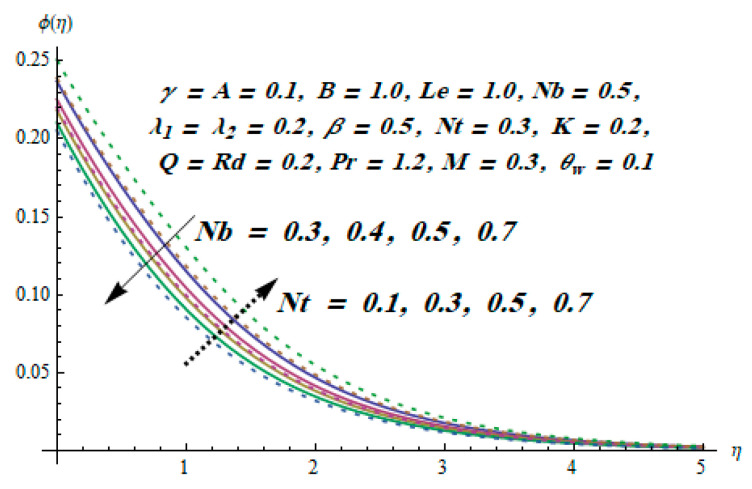
Effect of *Nt* and *Nb* on ϕ.

**Figure 9:**
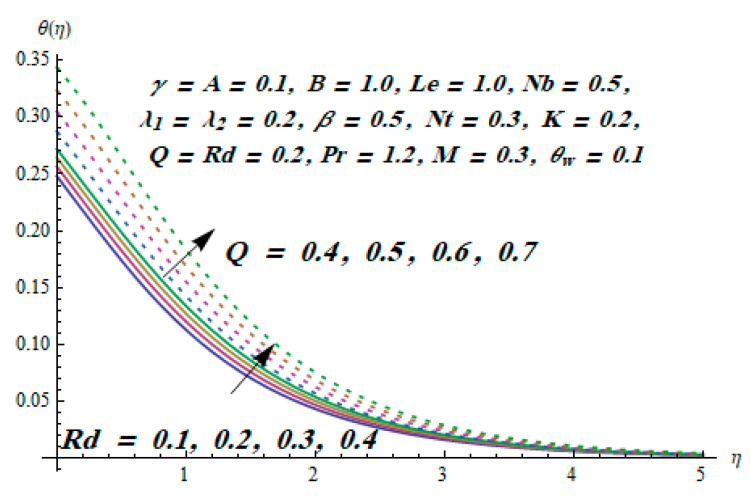
Effect of *Rd* and *Q* on θ.

**Figure 10:**
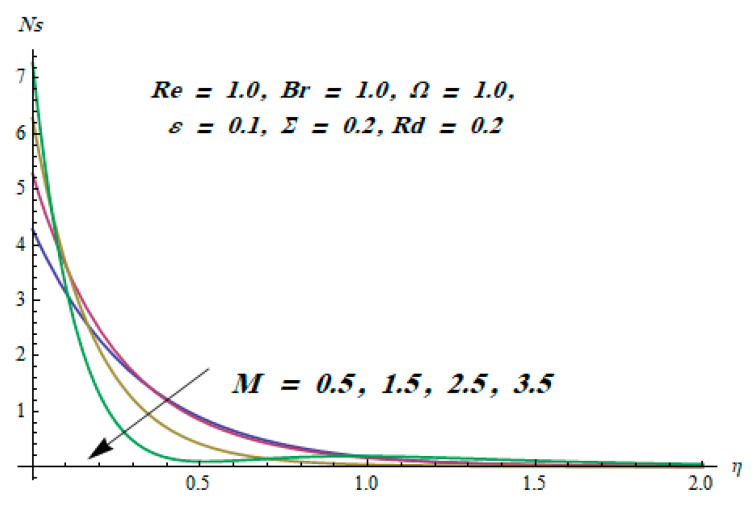
Effect of *M* on Ns.

**Figure 11:**
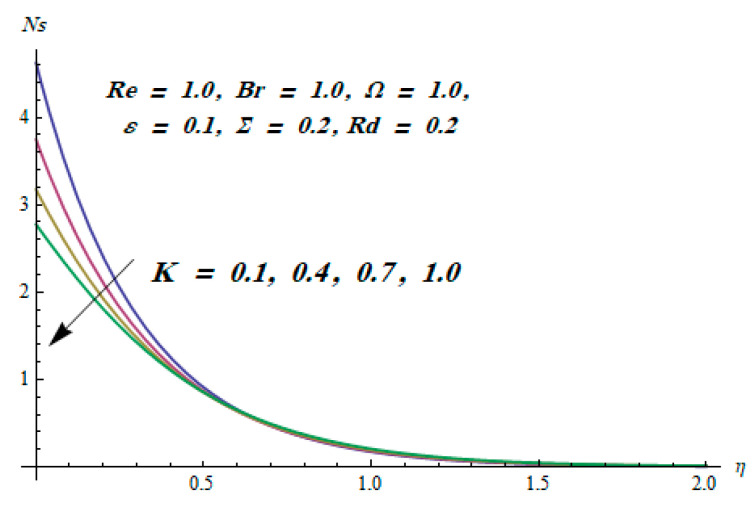
Effect of *K* on Ns.

**Figure 12:**
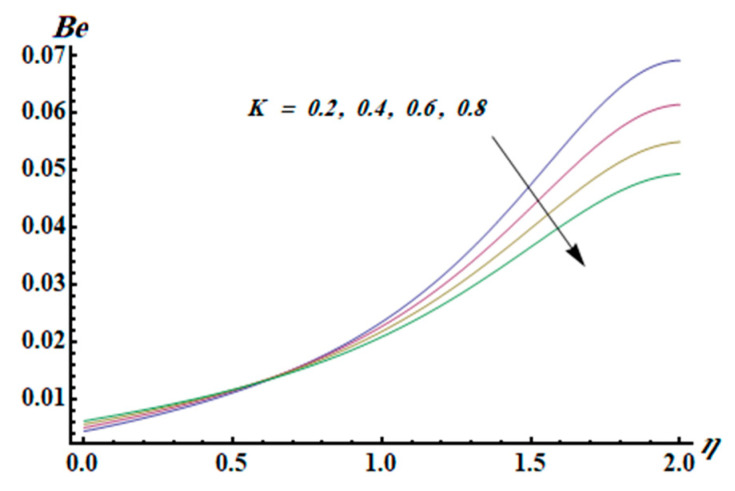
Effect of *K* on Be.

**Figure 13:**
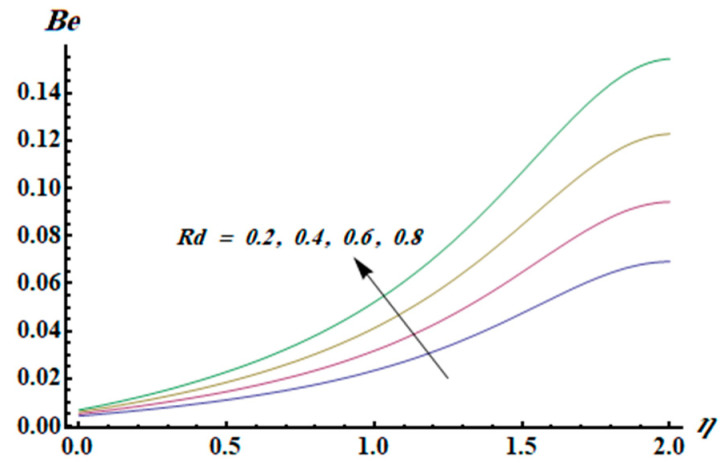
Effect of *Rd* on Be.

**Figure 14:**
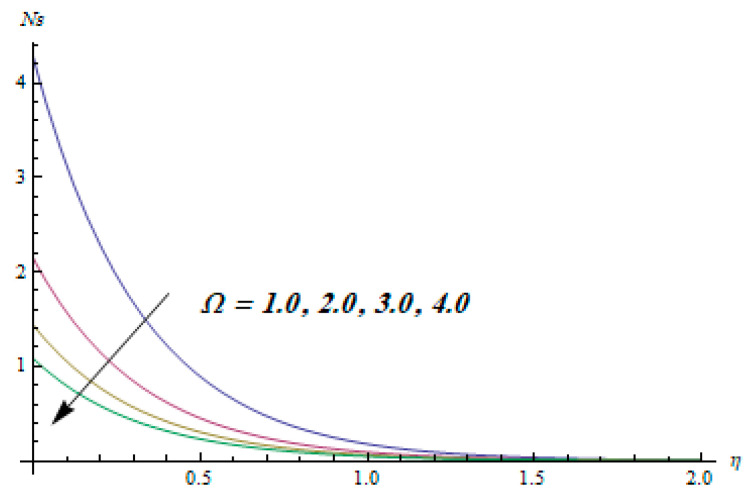
Effect of Ω on Ns.

**Figure 15:**
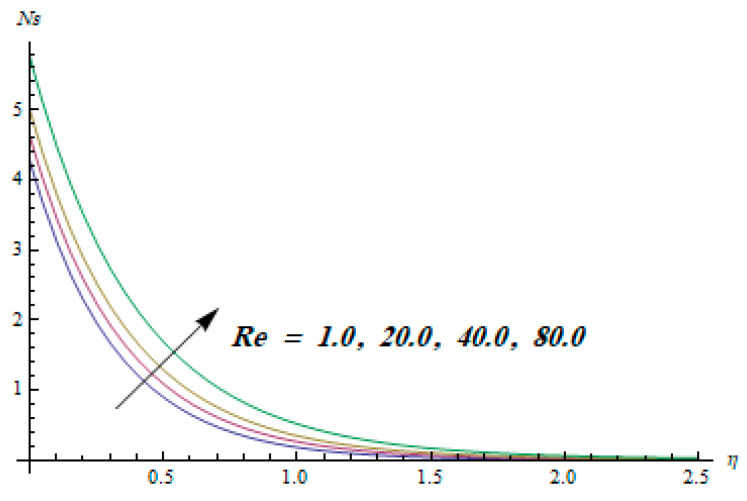
Effect of *Re* on Ns.

**Figure 16:**
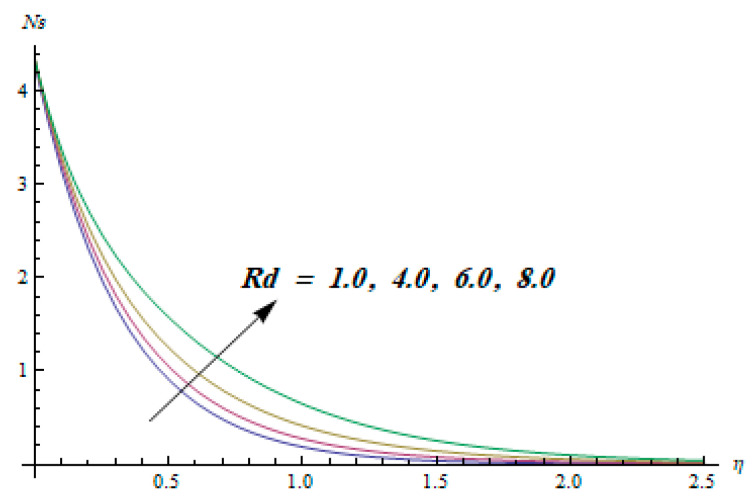
Effect of *Rd* on Ns.

**Figure 17:**
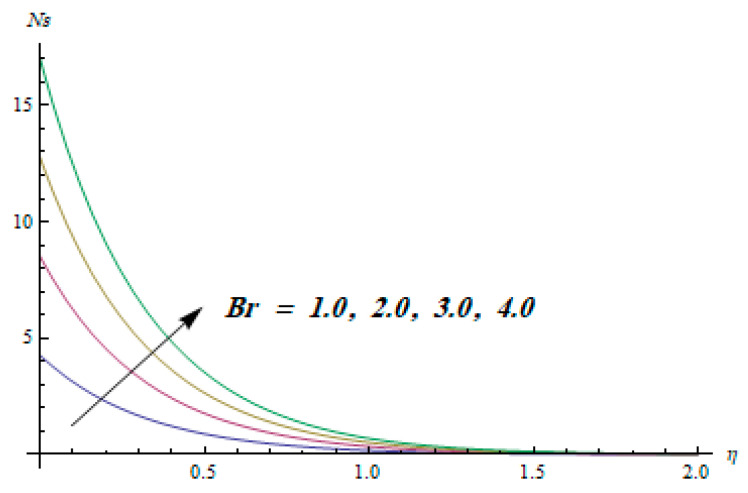
Effect of *Br* on Ns.

**Figure 18:**
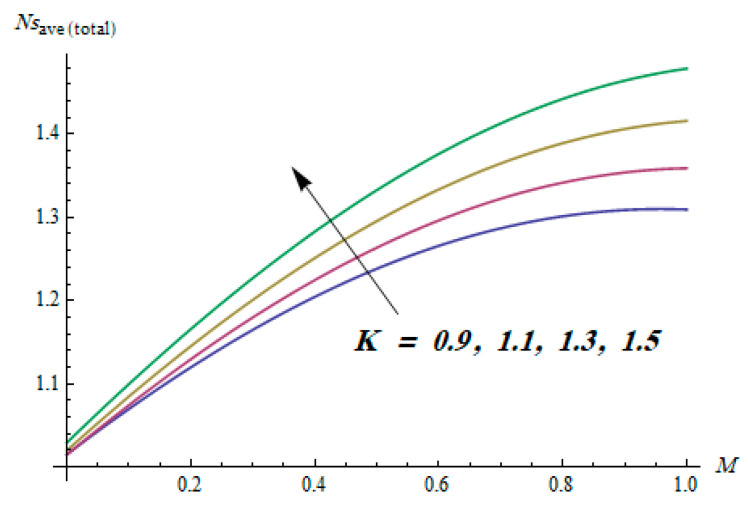
Effect of *M* and *K* on average entropy generation.

**Figure 19:**
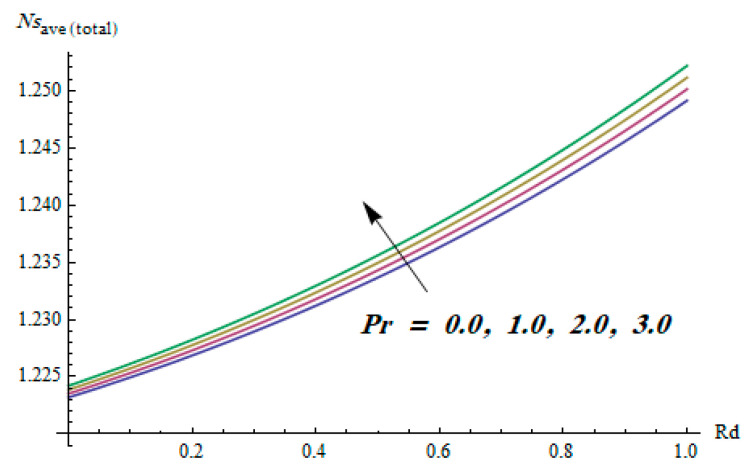
Effect of *Pr* and *Rd* on average entropy generation.

This section is dedicated to having a deep insight into changes of sundry parameters on involved profiles. The ranges of all parameters were selected via Turkyilmazoglu [51]. Figure 3 was drawn to show the impact of viscoelastic parameter K on both velocities. Both velocity distributions were declining functions of K. Augmented values of the K meant the development of tensile stress, which ultimately lowered both velocity components. The impression of magnetic parameter M on both velocity components is depicted in Figure 4. A diminishing behavior was witnessed for both velocity components. A strong Lorentz force was perceived due to the magnetic field, hindering the movement of the fluid flow. Thus, the reduction in velocity components was noticed. Figure 5 is outlined to grasp the impact of chemical reaction parameter γ and Prandtl number Pr on the concentration field. It was visualized that the rate of mass transfer was on the decline with augmented values of γ. Thus, the respected boundary layer was also enhanced. Smaller values of thermal diffusivity augmented the Pr and thereby lowered the concentration distributions. The outcome of temperature exponent A and Prandtl number Pr on temperature profile can be visualized in Figure 6. The Prandtl number is the quotient of momentum to thermal diffusivity. Smaller numbers for thermal diffusivity meant higher values of Pr, thus lowering the temperature. A similar effect was noted in the case of A. Figure 7 was illustrated to depict the influence of thermophoretic parameter Nt and Brownian motion parameter Nb on temperature field. It was comprehended that temperature was an increasing function of both parameters. This is because smaller particles were pushed toward the colder surface from the hotter one. Another reason for this phenomenon is that enhanced random movement of the fluid’s particles also became the main source of heightening the temperature of the fluid. To understand the influence of Brownian motion parameter Nb and thermophoresis parameter Nt on the concentration distribution, Figure 8 was plotted. Higher values of both parameters augmented the concentration profile. Smaller particles were pulled toward the colder region due to increased values of the thermophoretic parameter, thus lowering the concentration of the fluid. Figure 9 was drawn to grasp the impact of radiation parameter Rd and heat absorption parameter Q on the temperature field. In the experiment, the temperature rose with higher values of Rd. This is because growing values of *Rd* meant more emission of heat that ultimately raised the temperature of the fluid. Also, the higher estimates of Q produced more heat that also resulted in an increase in the fluid’s temperature. The variation of magnetic parameter *M* for the entropy generation Ns is presented in Figure 10. Initially, due to drag forces produced by the magnetic field, the energy was produced, which increased the entropy of the fluid. However, as it moved away from the plate, the influence of these forces reduced gradually and eventually lessened the effect of entropy as well. The effect of viscoelastic parameter K on entropy generation and the Bejan number Be is displayed in Figure 11 and Figure 12, respectively. Both the figures clearly indicate that the enhancement of values of K enhanced entropy generation as well as the Bejan number near the surface due to an increase in viscous effects. However, the opposite trend was noticed in both cases while moving away from the surface. In Figure 13, a relationship between the thermal radiation parameter Rd and dimensionless Bejan number Be is portrayed. An increment in thermal radiation parameter Rd enhanced the internal heat generation in the moving fluid, which caused the Bejan number to escalate. Figure 14, Figure 15, Figure 16 and Figure 17 were illustrated to show the impacts of dimensionless temperature difference Ω, Reynolds number *Re*, thermal radiation parameter *Rd*, and Brinkman number *Br* on the variation of local entropy generation number *Ns*, respectively. From Figure 14, it is revealed that as the temperature difference increased, a decrease in the entropy generation parameter was noticed. Larger values of Ω did not affect the viscous part of the entropy generation. However, only the thermal entropy generation segment was responsible for higher temperature gradients in the transverse direction. In Figure 15, Figure 16 and Figure 17, *Ns* was an increasing function of these three: *Re*, *Rd*, and *Br*. Figure 18 and Figure 19 were drawn to depict the influence of *M*, *K*, *Pr*, and *Rd* on average entropy *Ns_(avg)_*. From these figures, it was gathered that *Ns_(avg)_* was an escalating function of all four parameters. This meant irreversibility mounted as the group parameters grew.

Table 2 was initiated for the rate of heat and mass transfers versus varied involved parameters. It was noticed that the Nusselt number was a growing function of λ,Rd, Q and was a declining function of Nb, Le, Pr. Similarly, the Sherwood number decreased for the values of λ, Nb, Le, Pr, Rd, and increased for the values of Nt. Table 3 depicts the values of both skin friction coefficients along the x and y directions. It was gathered that both coefficients were growing functions of λ, M, and K. Table 4 was erected to check the reliability of the presented solution by comparing it to Liu et al. [52] in the limiting case. An excellent concurrence was obtained, confirming the reliability of the presented results.

## 6. Concluding Remarks

Entropy generation of 3D viscoelastic nanofluid fluid flow past an exponential stretched surface with nonlinear thermal radiation, chemical reaction, and magnetohydrodynamics was discussed analytically with the homotopy analysis method. The whole analysis was completed with the effect of heat generation and absorption supported by convective heat and mass boundary conditions. The main findings of the analysis are:

1. The velocity components were declining functions of the viscoelastic parameter.
2. The temperature field improved with an increase in radiation parameter.
3. Thermophoresis and Brownian motion parameters had an opposite effect on concentration distribution.
4. With growing values of the magnetic parameter, both velocity components declined.
5. The Bejan number is an increasing function of the thermal radiation parameter.
6. Entropy generation decreased for escalating values of the temperature difference parameter.

## Figures and Tables

**Table 1:** Series solution convergence for varied orders of approximations when λ = 0.1, *Nb* = 0.5, *Nt* = 0.3, *K* = 0.2, *M* = 0.3, *Le* = 1, *Pr* = 1.2, *Rd* = 0.2, *A* = *B* = 0.1.

Order of Approximation	$-f^{\prime \prime }\left(0\right)$	$-g^{\prime \prime }\left(0\right)$	$-\theta^{\prime }(0)$	−ϕ(0)
1	1.19588	0.12037	0.16113	0.16180
3	1.37115	0.13887	0.15382	0.15745
7	1.42903	0.14518	0.14911	0.15586
10	1.45762	0.14852	0.14206	0.15476
13	1.46000	0.14883	0.13937	0.15471
14	1.46000	0.14883	0.13798	0.15460
15	1.46000	0.14883	0.13796	0.15460
18	1.46000	0.14883	0.13796	0.15460

**Table 2:** Values of local Nusselt and Sherwood numbers for involved parameters.

*λ*	*Nb*	*Nt*	*Le*	*Pr*	*M*	*K*	*Rd*	*A*	*Q*	$-{\left(\frac{Re}{2}\right)}^{-\frac{1}{2}}Nu_{x}.$	$-{\left(\frac{Re}{2}\right)}^{-\frac{1}{2}}Sh_{x}$
0.1	-	-	-	-	-	-	-	-	-	0.13878	0.12199
0.2	-	-	-	-	-	-	-	-	-	0.1388	0.12157
0.5	-	-	-	-	-	-	-	-	-	0.1421	0.12032
-	0.5	-	-	-	-	-	-	-	-	0.13878	0.12199
-	1.0	-	-	-	-	-	-	-	-	0.13772	0.11917
-	1.5	-	-	-	-	-	-	-	-	0.13770	0.11823
-	-	0.0	-	-	-	-	-	-	-	0.13878	0.11635
-	-	0.2	-	-	-	-	-	-	-	0.13878	0.12199
-	-	0.5	-	-	-	-	-	-	-	0.13878	0.12576
-	-	-	1.0	-	-	-	-	-	-	0.14447	0.12981
-	-	-	1.5	-	-	-	-	-	-	0.13878	0.12199
-	-	-	2.0	-	-	-	-	-	-	0.12572	0.11010
-	-	-	-	1.0	-	-	-	-	-	0.13878	0.12199
-	-	-	-	1.2	-	-	-	-	-	0.13775	0.08889
-	-	-	-	1.5	-	-	-	-	-	0.13774	0.06499
-	-	-	-	-	0.0	-	-	-	-	0.13878	0.12199
-	-	-	-	-	0.2	-	-	-	-	0.13878	0.12199
-	-	-	-	-	0.3	-	-	-	-	0.13878	0.12199
-	-	-	-	-	-	0.0	-	-	-	0.13878	0.12199
-	-	-	-	-	-	0.02	-	-	-	0.13878	0.12199
-	-	-	-	-	-	0.04	-	-	-	0.13878	0.12199
-	-	-	-	-	-	-	0.2	-	-	0.13878	0.12199
-	-	-	-	-	-	-	0.4	-	-	0.14392	0.12146
-	-	-	-	-	-	-	0.5	-	-	0.14480	0.12123
-	-	-	-	-	-	-	-	0.1	-	0.13878	0.12199
-	-	-	-	-	-	-	-	0.5	-	0.13878	0.12199
-	-	-	-	-	-	-	-	0.7	-	0.13878	0.12199
-	-	-	-	-	-	-	-	-	0.2	0.13878	0.12199
-	-	-	-	-	-	-	-	-	0.4	0.14962	0.12199
-	-	-	-	-	-	-	-	-	0.5	0.15679	0.12199

**Table 3:** Values of skin friction coefficients for involved parameters.

*λ*	*M*	*K*	$-{\left(\frac{Re}{2}\right)}^{1/2}C_{f_{x}}$	$-{\left(\frac{Re}{2}\right)}^{1/2}C_{f_{y}}$
0.1	-	-	1.6768	0.2237
0.2	-	-	1.7698	0.4089
0.5	-	-	2.0571	1.0804
-	0.3	-	1.6768	0.2237
-	0.5	-	1.7422	0.2325
-	1.0	-	2.0212	0.2697
-	-	0.02	1.6768	0.2237
-	-	0.03	1.9607	0.2675
-	-	0.04	1.8168	0.3138

**Table 4:** Comparison of present values to Liu et al. [52] in the limiting case when K=Nb=Nt=Rd=Q=M=0 (also, values of the convective boundary were neglected).

*β*	*Pr*	*A*	Liu et al. [52]	Present Study
0.0	0.7	0.0	−0.42583804	−0.4258120
		2.0	−1.02143617	−1.0214514
		5.0	−1.64165922	−1.6416620
0.25	0.7	0.0	−0.47609996	−0.4761032
		2.0	−1.14199997	−1.1420014
		5.0	−1.83543073	−1.8354210
0.50	0.7	0.0	−0.52154103	−0.5215267
		2.0	−1.25099820	−1.2509991
		5.0	−2.01061361	−2.0106021
0.75	0.7	0.0	−0.56332861	−0.5633148
		2.0	−1.35123246	−1.3512221
		5.0	−2.17171091	−2.1717006
1.0	0.7	0.0	−0.60222359	−0.6022167
		2.0	−1.44452826	−1.4445214
		5.0	−2.32165661	−2.3216340

## Nomenclature

*a, b, c, d, e*	Dimensional constants
*η*	Similarity variable
*A*	Temperature exponent
*B*	Concentration exponent
*Be*	Bejan number
*β*_0_	Magnetic field strength
*C*	Concentration of fluid
*C_f_*	Skin friction
*c_p_*	Specific heat
*C_w_*	Concentration on wall
*C*_∞_	Ambient concentration
*C*_0_	Reference concentration
*Br*	Brinkman number
*D_B_*	Brownian diffusion coefficient
*D_T_*	Thermophoretic diffusion coefficient
*f, g*	Dimensionless velocities
$\rho c_{f}$	Effective heat capacity of nanoparticles
*k*	Thermal conductivity
*K*	Viscoelastic parameter
*k_o_*	Elastic parameter
*K**	Mean absorption coefficient
*α*	Effective heat capacity of fluid
*Le*	Lewis number
*Nb*	Brownian motion parameter
*Nt*	Thermophoresis parameter
*Nu_x_*	Nusselt number
*M*	Magnetic parameter
*Pr*	Prandtl number
*Q*	Heat absorption
*Rd*	Thermal radiation parameter
*Re*	Reynolds number
*S_G_*	Volumetric entropy generation
*Nu_x_*	Local Nusselt number
*N_S_*	Entropy generation rate
*C_fx_, C_fy_*	Skin friction coefficients
*Sh_x_*	Sherwood number
*T*	Temperature of fluid
*T_w_*	Wall temperature
$U_{0},V_{0},T_{0},C_{0}$	Constants
*T*_∞_	Ambient temperature
*U_e_*	Stretching velocity
*U_w_*	Linear stretching velocity
*(u, v, w)*	Velocity components
*(x, y, z)*	Coordinate axes
*M*	Hartmann number
ν	Kinematic viscosity
λ_1_	Relaxation time
Λ_2_	Ratio of relaxation to retardation time
*ρ*	Density of fluid
*σ*	Electrical conductivity
*σ**	Stefan–Boltzmann constant
*μ*	Dynamic viscosity
*τ*	Ratio of nanoparticle
*τ_w_*	Skin friction coefficient
Ω	Dimensionless temperature difference
*ε*	Dimensionless nanoparticle volume difference
*Σ*	Nanoparticle mass transfer parameter
*θ*	Dimensionless temperature
*ϕ*	Dimensionless concentration
*α*_1_	Normal stress moduli
*K_c_*	Chemical reaction coefficient
L	Reference length
